# Lysophosphatidic acid-mediated enhancement of porcine oocyte chemical enucleation for somatic cell nuclear transfer

**DOI:** 10.5713/ab.260269

**Published:** 2026-06-01

**Authors:** Chi-Hong Wong, Shu-Ping Chang, Shu-Hui Peng, Jyh-Cherng Ju, Yu-Ten Ju, Pin-Chi Tang, San-Yuan Huang, Ching-Fu Tu

**Affiliations:** 1 Division of Animal Technology, Animal Technology Research Center, Agricultural Technology Research Institute, Hsinchu, Taiwan; 2 Department of Animal Science, National Chung Hsing University, Taichung, Taiwan; 3 Graduate Institute of Biomedical Sciences, China Medical University, Taichung, Taiwan; 4 Translational Medicine Research Center, China Medical University Hospital, Taichung, Taiwan; 5 Department of Animal Science and Technology, National Taiwan University, Taipei, Taiwan; 6 The iEGG and Animal Biotechnology Center, National Chung Hsing University, Taichung, Taiwan; 7 Professional Master Program of Agricultural Business Management, National Chung Hsing University, Taichung, Taiwan

**Keywords:** Chemical Enucleation, Lysophosphatidic Acid, Nocodazole, Reconstituted Porcine Embryo, Somatic Cell Nuclear Transfer

## Abstract

**Objective:**

Protocols for somatic cell nuclear transfer (SCNT) remain technically challenging and inefficient. Demecolcine and nocodazole (Noc) have been used for chemically assisted enucleation to improve cloning efficiency; however, the procedure continues to be labor-intensive. This study aimed to use lysophosphatidic acid (LPA) to enhance Noc-mediated chemical enucleation (CE), thereby simplifying the SCNT procedure.

**Methods:**

Porcine MII oocytes were subject to CE by incubation with Noc and various concentrations of LPA for 1 h. Actin signals were evaluated to determine the optimal LPA. After treatment with Noc and 25 μM LPA for 1 h, spindle reformation was assayed following subsequent washout. Noc/LPA CE-treated oocytes were used for SCNT via direct microinjection of donor cells into the ooplasm. Reconstructed embryos were recovered for 2 h in PZM-5 prior to activation with ionomycin and *N,N,N′,N′*-tetrakis (2-pyridylmethyl) ethylenediamine. Activated reconstructed embryos were cultured in 6-DMAP for 4 h, then in chaetocin for an additional 20–24 h to promote demethylation and acetylation. Cleavage and blastocyst rates were evaluated on Day 2 and Day 6, respectively.

**Results:**

The culture of the matured oocytes in Noc/LPA for 1 h, the percentage of oocytes exhibiting actin signals increased significantly from 12.5% to 87.6% with the addition of 25 μM LPA. Although spindles reformed in 59.0% of oocytes 1 h after Noc/LPA washout, 83.9% of oocytes retained spindles enclosed by actin rings; enabling the direct microinjection of donor cells into the ooplasm within 30 min. Blastocyst development rates currently vary from 9.5% to 16.6% after 6 days of culture in PZM-5.

**Conclusion:**

Although the blastocyst rate requires further improvement, we have shown that enucleation of MII pig oocytes can be successfully achieved via Noc/LPA treatment. This protocol simplifies the SCNT procedure while ensuring consistent blastocyst development in pig *in vitro* maturation oocytes.

## INTRODUCTION

Gene editing (GE) through CRISPR/Cas9 approach has led to a novel way for pig breeding, with most of the efforts combining somatic cell nuclear transfer (SCNT) with GE donor cells [1]. However, SCNT currently remains a highly demanding technique that is both tedious and inefficient. Standard SCNT manipulations involve the use of *in vivo* or *in vitro* maturation (IVM) oocytes, mechanical enucleation, sub-zonal microinjection of donor cells, electronic fusion and activation, and chemical treatment or mRNA injection to improve reprogramming of reconstructed embryos. While many detailed strategies to improve SCNT efficiency have been discussed [2], none of them includes direct chemical enucleation (CE).

Although chemically assisted (CA) enucleation (CAE) has been explored [3], and nocodazole (Noc) was used to facilitate CAE in rat oocytes [4], Yin et al. [5] were the first to utilize demecolcine (Dem) to facilitate CAE of rabbit MII chromosomes by inducing their protrusion from the oocyte cytoplasm. This method was subsequently applied to generate live cloned pigs [6] and bovine fetuses [7]. When comparing the effects of Dem and Noc, Kawakami et al. [8] found no significant differences in cleavage rates, blastocysts (BC) formation rates, or BC cell numbers. However, live-born cloned piglets [8] and live healthy cloned calf [9] were produced using the Dem-treated group.

An optimized protocol for goat oocyte CAE was established by Lan et al. [10], which significantly improved blastocyst formation compared with blind enucleation but showed little difference in the generation of cloned goats. Their report also elucidated several mechanisms of CAE: While protrusions could persist for 2 h, most disappeared within 30 min after cytochalasin B (CB) treatment. Additionally, both the formation of an actin-rich ring around the protruding condensed chromosomes and mitogen-activated protein kinase (MAPK) activity were significantly increased [10]. Furthermore, Costa-Borges et al. [11] demonstrated that distinct antimitotic agents induced different types of protrusions in oocytes across species. These were classified as Type A or Type B protrusion; Type B was characterized by a more pronounced membrane projections appearing near enucleated oocytes [11].

The mechanism of chemically induced cytokinesis for assisted enucleation involves the interplay among maturation-promoting factor (MPF), MAPK, and Rho GTPase [12]. This interaction governs the assembly of the actomyosin ring, which is promoted by MG132 and inhibited by Y27632 (a ROCK inhibitor). The authors concluded that MPF-mediated activation of MAPK is the initial step, followed by a decrease in MPF activity that triggers ring contraction via RhoA activation. This implies that altering MPF, MAPK, or ROCK activity directly affects the efficiency of CE.

Lysophosphatidic acid (LPA), a ROCK activator [13], can organize the actin cytoskeleton by activating the Rho/ROCK signaling pathway [14]. Theoretically, LPA is capable of improving the reliability of the Dem- or Noc-mediated CE.

We aimed to combine Dem or Noc with LPA and/or ionomycin (Ion)/cycloheximide (CHX) to achieve more efficient CE. Furthermore, we sought to simplify the SCNT procedure by directly microinjecting the donor cells into the CE ooplasm. Additionally, we designed experiments to adjust the timing of 6-DMAP treatment, either during or after the recovery of reconstructed embryos—to streamline the overall SCNT process.

## MATERIALS AND METHODS

### Chemicals and reagents

Most chemicals were purchased from Sigma-Aldrich, including LPA (L7260). Additional, reagents were obtained from the following sources: Medium 199 (11150-059; Gibco); PMSG (HOR-272; ProSpec); hCG (230734; Merck); Alexa Fluor 594 Phalloidin (A12381; Life Technologies); PZM-5 (CK024; Cosmo Bio); and FBS (SH30084.03; HyClone).

### Donor cell preparation

The porcine fibroblast cells used in this study were derived from ear tissue biopsies and established in our laboratory including various sources: a β-actin/hHO-1 and β-actin/GFP dual transgenic male neonate (L20-01), a female minipig (mini-4; [15]), and an adult elite boar (L277-10; [16]). Donor cells were maintained in DMEM, and passages 5 through 7 (P5-P7) were utilized for SCNT. Cells were selected for SCNT based on morphology and size; specifically, spherical and small cells with a diameter of 11–13 μm were preferred. No serum starvation was performed prior to SCNT.

### Ovarian oocytes collection

Porcine ovaries were harvested from a local abattoir and transported to the laboratory within 1 h in a container maintained at approximately 35°C. During transit, the ovaries were immersed in 0.9% (w/v) saline supplemented with 10× streptomycin/penicillin G. Cumulus-oocyte-complexes (COCs) were aspirated from follicles ranging from 3 to 8 mm in diameter using an 18-gauge needle attached to a 10- or 20-mL syringe. Only COCs possessing at least three compact layers of cumulus cells were selected for IVM.

### Porcine oocytes *in vitro* maturation

Approximately 400 COCs were used for each IVM session. Groups of 20–25 COCs were cultured in a 60 μL droplets of IVM medium under mineral oil. The IVM medium was supplemented with 10 IU/mL PMSG, 10 IU/mL hCG, 20 ng/mL EGF, 20 ng/mL non-essential amino acids, 100 μg/mL cysteine, 10 nM melatonin, 50 μg/mL Vit. C, and 10% (v/v) porcine follicular fluid. The COCs were cultured at 38.5°C in a 5% CO_2_ atmosphere for approximately 22 h, after which they were transferred to the same culture medium without PMSG and hCG for an additional 22–24 h. Following maturation, all well-expended COCs were collected, and cumulus cells were removed by treatment with 0.1% hyaluronidase. The oocytes were then placed in D-PBS containing 0.025 M sucrose (330 mOsm), and those exhibiting a visible first polar body (metaphase II, MII) were selected for subsequent trials.

### Chemical enucleation and assessment of porcine matured oocytes

After 43–46 h of IVM, MII oocytes were further cultured for 1 h in D-PBS (without Ca^2+^ and Mg^2+^) containing 3 μg/mL Noc and various concentration of LPA (0, 10, 25 and 50 μM). To visualize microtubules, oocyte samples were fixed in 4% paraformaldehyde for 2 h and permeabilized in 0.2% Triton X-100 for additional 15 min. Samples were incubated overnight (~12 h) with a mixture of primary mouse monoclonal anti–α/β-tubulin antibodies, followed by with a FITC-conjugated rabbit anti-mouse IgG secondary antibody for 1 h. Cortical microfilaments and DNA were stained with 1.5 IU/mL Alexa Fluor 594 phalloidin and 5 μg/mL Hoechst 33342, respectively, to assess actin formation around the protruding meiotic nucleus and enucleated oocytes.

Following 1 h of treatment with 3 μg/mL Noc and 25 μM LPA, oocytes were transferred to PZM-5 medium and cultured for 0, 1, 2 and 4 h. The oocytes were then fixed, permeabilized, and stained according to the previously described procedures to assess the reformation of the spindle and the positioning of chromosome within the actin ring.

To evaluate the effects of activation using Ion (15 μM) and/or CHX (10 μg/mL) on CE, MII oocytes were cultured in either 0.4 μg/mL Dem or 3 μg/mL Noc, each supplemented with 25 μM LPA. Ion activation was administered either 5 min before or concurrently with Noc/LPA or Dem/LPA enucleation. In the combined treatment groups, 10 μg/mL CHX treatment was added during the 60-min enucleation period (Noc/LPA/Ion/CHX or Dem/LPA/Ion/CHX). Actin and chromosomal assessments were conducted as described above.

### Somatic cell nuclear transfer and culture of reconstructed embryos

**Figure 1:**
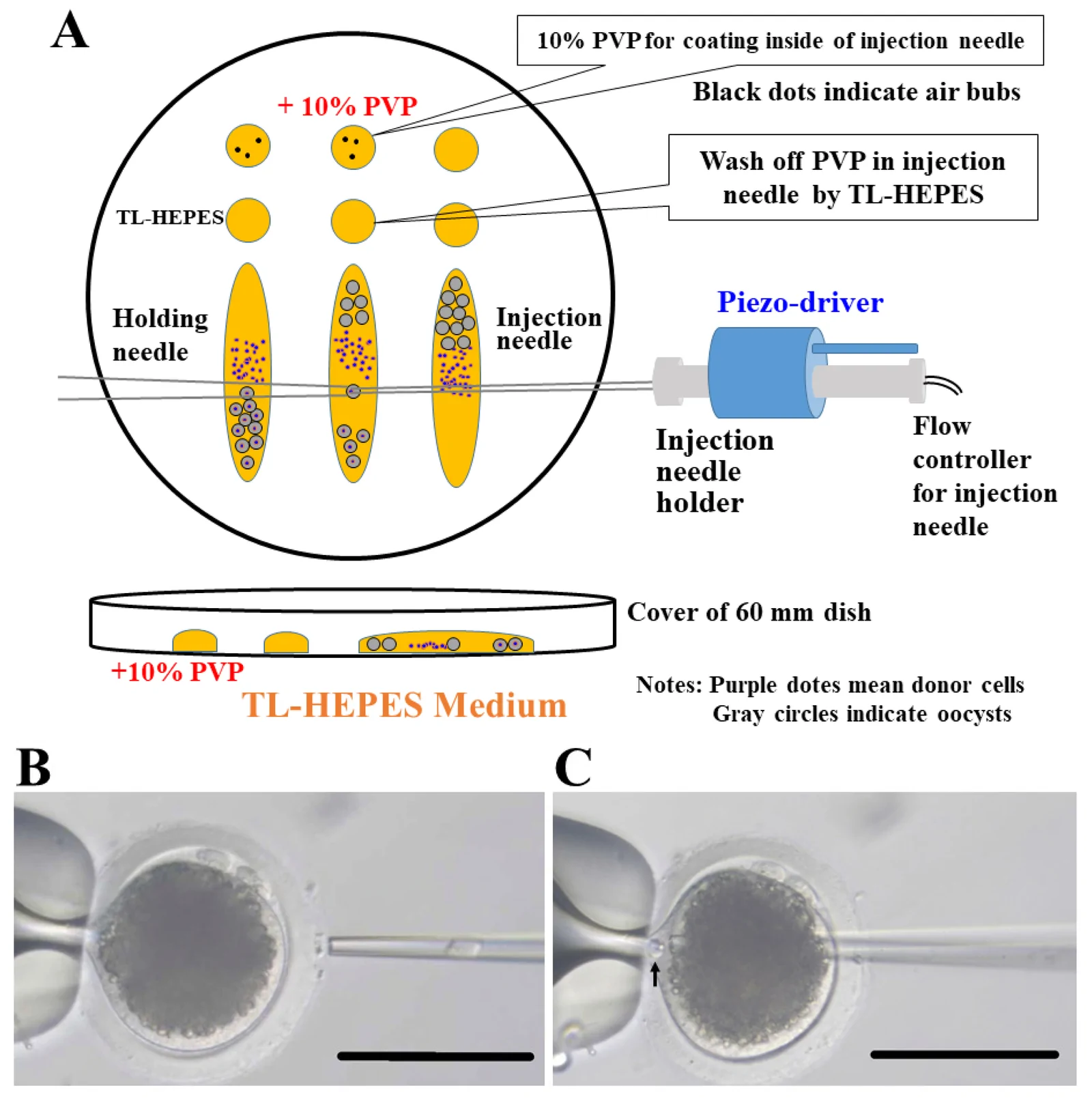
Preparation and conduction of direct somatic cell nuclear transfer into chemical enucleated porcine oocytes. (A) Schematic of micromanipulation setup: Step 1: Submerge the tip of the injection needle deep into the 10% Polyvinylpyrrolidone (PVP) droplet in the upper section of the manipulation chamber. Aspirate and expel the PVP solution 3–4 times to stabilize flow, then expel the PVP from the tip. Step 2: Move the injection needle into TL-Hepes droplet (middle section) and use suction to fill the distal portion of the needle. Step 3: Transfer the needle to the bottom droplet containing the ooplasm and donor cells. Capture a donor cell and perform direct microinjection into the ooplasm, using a Piezo-driver to penetrate zona pellucida and plasma membrane. (B) A chemically enucleated oocyte before donor cell injection. (C) A donor cell (indicated by black arrow) injected into the enucleated oocyte. Black bars represent 100 μm.

Prior to SCNT, MII oocytes were cultured for 1 h in Ca^2+^/Mg^2+^-free D-PBS supplemented with 3 μg/mL Noc and 25 μM LPA to induce chromosomal enucleation. The SCNT micromanipulation setup—including oocytes, donor cells, 10% PVP/TL-Hepes, and TL-Hepes medium—is depicted in Figure 1. A microinjection needle was operated via a Piezo-micromanipulator controller (OMAS-CT150; Prime Tech). In each session, donor cells with diameters ranging from 11–13 μm were selected and microinjected into approximately 50 enucleated oocytes; each session was completed within 30 min.

In the first SCNT experiment, L20-01 and mini-4 fibroblasts were used to establish SCNT procedures by direct microinjecting donor cells into the ooplasm of Noc/LPA treated oocytes. The reconstructed embryos were recovered in PZM-5 supplemented with 5 μg/mL CB, 1 mM 6-DMAP, and 5 μM Y27632 (a ROCK inhibitor) for 3 h, followed by chemical activation.

In the second SCNT experiment, L277-10 fibroblasts were used as donor cells. We compared two 6-DMAP treatment protocols for the reconstructed embryos: The embryo-treated before activation (EBA) group, in which embryos were recovered in PZM-5 supplemented with 2 mM 6-DMAP for 3 h prior to chemical activation; and the embryo-treated after activation (EAA) group, in which embryos were recovered in PZM-5 for 2 h, chemically activated, and subsequently treated with 2 mM 6-DMAP for 4 h.

Chemical activation of all reconstructed embryos was performed using 15 μM Ion for 5 min, followed by 5 μM TPEN for 15 min, according to the protocol reported by de Macedo et al. [17]. Following activation and 6-DMAP treatment, the reconstructed embryos were cultured in PZM-5 medium supplemented with 0.5 nM chaetocin for 20–24 h, then transferred to fresh PZM-5 for further *in vitro* culture (IVC). To evaluate survival and developmental competence, the lysis, cleavage and blastocyst formation rates were assessed at 24, 48, and 144/168 h post-activation, respectively. During the IVC period, the PZM-5 medium—supplemented with 50 μg/mL Vit. C, 10 nM melatonin, 1.69 mM arginine, and 3 mg/mL BSA—was refreshed every other day, and 10% FCS was added on day 5.

### Assessment of cell number of blastocysts

After being cultured in PZM-5 for 6 and 7 days, the reconstructed embryos were examined using an Olympus IX71 inverted microscope (IX71; Olympus). To determine the total cell count, embryos that reached the blastocyst stage by day 7 were fixed with 4% paraformaldehyde and stained with Hoechst 33342. Cell numbers were then recorded using an Olympus BX50 fluorescence microscope (BX50; Olympus) under UV excitation.

### Statistical analysis

Data normality was assessed with the Shapiro–Wilk test. Normally distributed data were analyzed using one-way ANOVA with Tukey’s post hoc test. Spindle formation data were analyzed with a binomial generalized linear model, and pairwise comparisons were conducted using Fisher’s exact test. Statistical analyses were performed with R software v4.3.1 (R Foundation for Statistical Computing). A p-value of <0.05 was considered significantly different. The t-test analysis was conducted by Excel software (Microsoft).

## RESULTS

### Lysophosphatidic acid potentiates nocodazole- or demecolcine-induced enucleation in porcine oocytes

**Figure 2:**
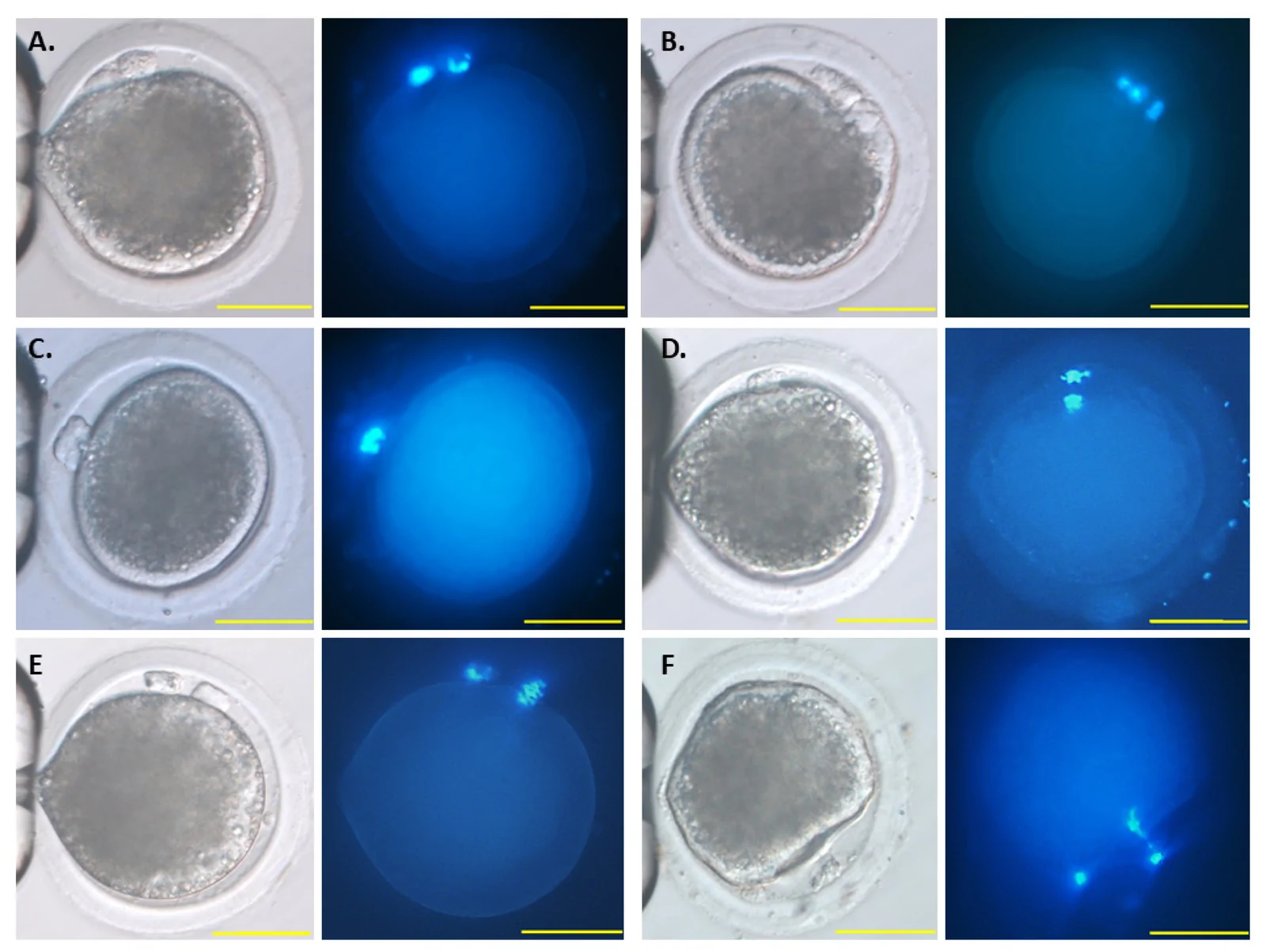
Chemical enucleation of porcine oocytes. (A–C) Oocytes show successful and complete enucleation with two, three and one polar bodies, respectively; (D) A metaphase II (MII) oocyte that failed to undergo enucleation. (E, F) Oocytes exhibiting two polar bodies optically, but with the second polar body incompletely expelled (indicated by white arrows). Yellow bars represent 50 μm.

We aimed to establish an optimized CE protocol for SCNT. Using current methods with Dem or Noc alone, we observed two polar bodies in 79.4±5.3% or 82.1±7.1% of oocytes, respectively (Supplement 1). However, during aspiration, some polar bodies remained adhered to the oocyte plasma membrane. Detection of residual chromosomes in the ooplasm confirmed that enucleation was incomplete (Figure 2), suggesting these chemicals primarily serve as enucleation aids.

**Figure 3:**
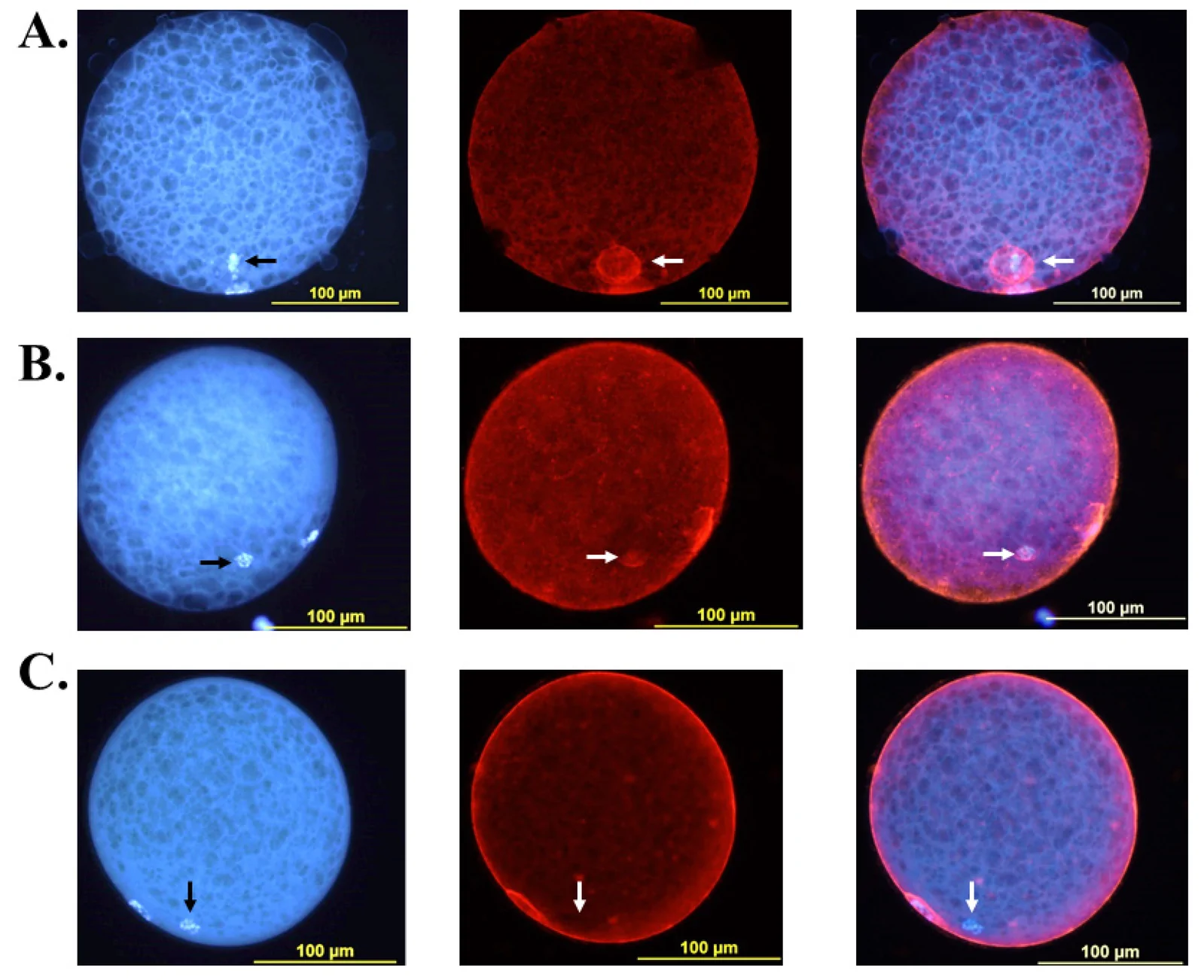
Lysophosphatidic acid (LPA) potentiates nocodazole-induced chemical enucleation of porcine oocytes. (A) Completely enucleated with a strong actin signal enclosing the entire chromosome set. (B) Completely enucleated with a weak actin signal enclosing the entire chromosome. (C) Metaphase II (MII) oocyte that failed to undergo enucleation. Arrows indicate chromosomes.

To improve the efficiency, we attempted to potentiate Noc and Dem for porcine oocytes enucleation by adding LPA. Firstly, Noc was used alone to determine the optimal LPA concentration. As shown in Table 1, 25 μM LPA induced the complete extrusion of the second polar body in 60.2±5.5% of oocytes, which was confirmed by a strong actin signal (Figure 3). When including oocytes with weak actin signal, 87.6±3.8% of oocytes were considered successfully enucleated and deemed suitable for direct donor cell microinjection for SCNT. Furthermore, since the enucleation efficiency of the Dem/LPA combination (Supplement 2) was not superior to Noc/LPA combination, we utilized the Noc/LPA CE method for subsequent SCNT procedures.

**Figure 4:**
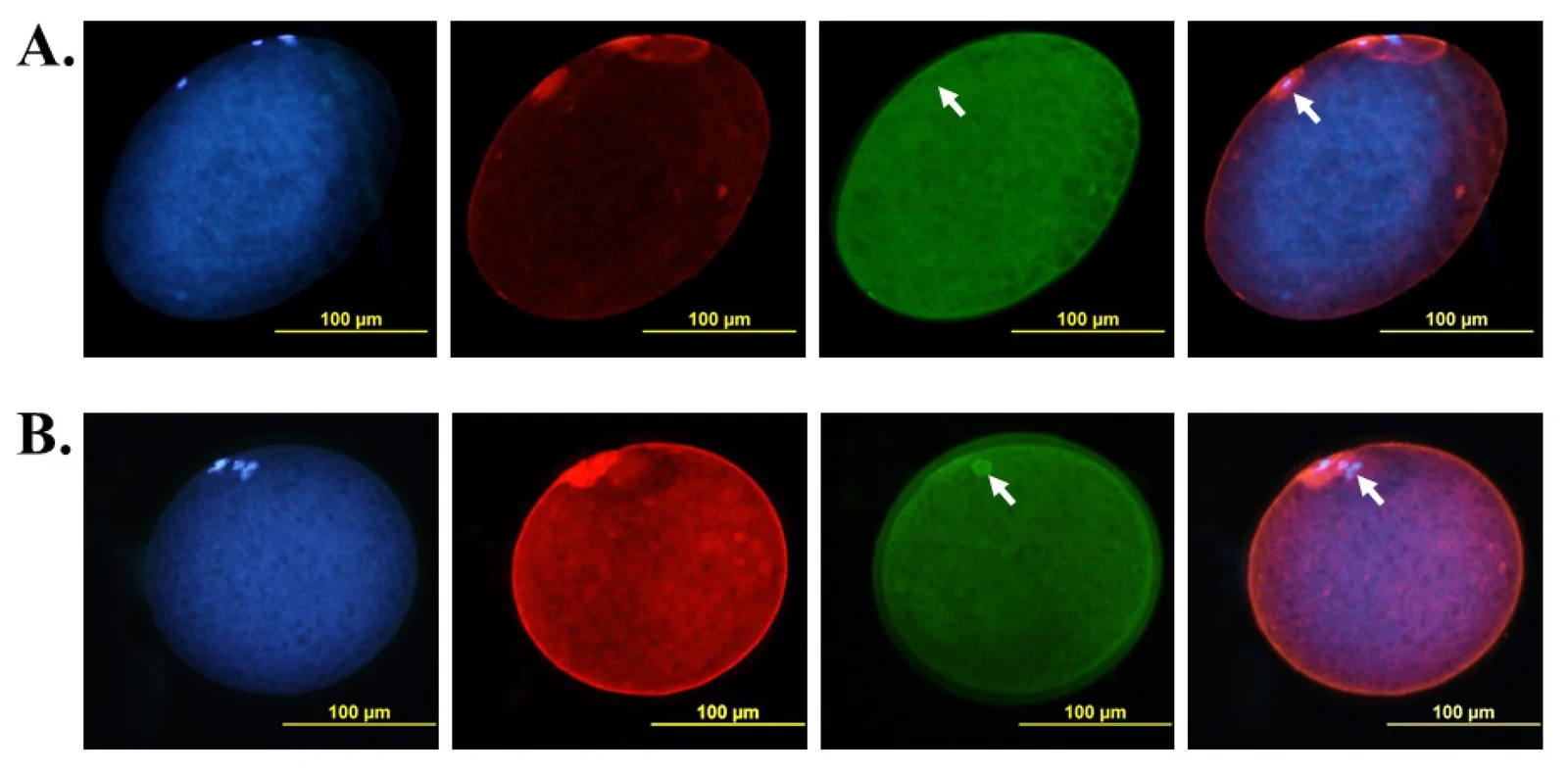
Spindle reformation following the removal of nocodazole (Noc) and lysophosphatidic acid (LPA) during chemical enucleation. The entire chromosomal set remains enclosed by actin; (A) No spindles have reformed, while (B) Spindles have reformed after the removal of Noc and LPA for 1 h. White arrows indicate enucleated chromosomes.

We further evaluated the effect of Noc/LPA on the meiotic spindle by washing out the reagents and culturing the CE oocytes in PZM-5 for 0, 1, 2, and 4 h. As detailed in Table 2, results from the 1- to 4-h recovery periods revealed that 40.7±23.1% to 44.5±17.8% of nuclei remained without a chromosomal spindle (Figure 4A). Conversely, 55.5±17.8% to 63.1±22.0% of oocytes exhibited spindle reformation (Figure 4B). Notably, even when the spindle reformed, it remained enclosed by an actin ring (Figure 4B); specifically, 83.9±7.3% and 88.1±7.4% of oocytes retained the actin signal after 1 h and 2 h of washout, respectively (Table 2). In fact, the meiotic spindle may reform as early as 0.5 h after Noc/LPA washout while remaining enclosed within the actin ring (Supplement 3). These results indicated that enucleation was successfully achieved in those instances. Based on these observations, we propose that SCNT via direct microinjection into the CE ooplasm is most effective when completed within 30 min of reagent removal, as the microinjection process itself may further assist in extrusion of any remaining chromosomes.

### Effect of activation on chemical enucleation

During natural fertilization, sperm penetration activates the oocyte and triggers the expulsion of the second polar body. We examined whether activation with Ion or Ion/CHX could enhance the efficiency of CE. While combining Dem/LPA or Noc/LPA with Ion/CHX achieved enucleation rates of 83.7±6.0% and 85.0±2.1%, respectively—defined by the presence of actin signals (Table 3)—these results were not significantly superior to Dem/LPA (76.8±5.6%) or Noc/LPA only (78.5±2.1%) alone (p>0.05). Consequently, the marginal improvement did not justify this strategy. Furthermore, Ion/CHX may induce cell cycle asynchrony between the donor cell and ooplasm and introduces uncertainty regarding the necessity of a second activation following donor cell injection. For these reasons, we utilized only the Noc/LPA treatment for CE and SCNT.

### Direct microinjection of somatic cells into chemically enucleated ooplasms

**Figure 5:**
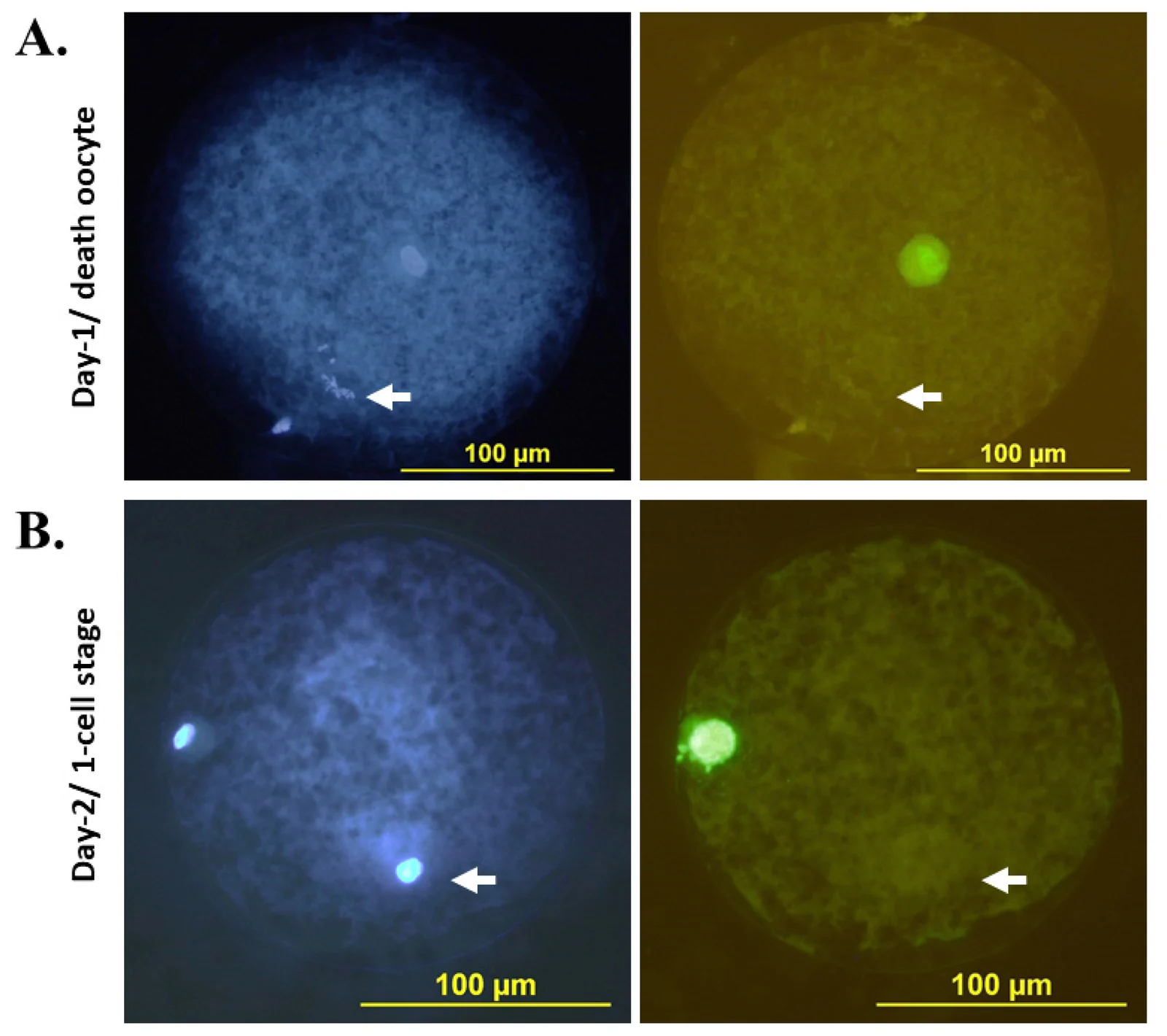
Assessment of degenerate and arrested reconstructed embryos produced via chemical enucleation and direct microinjection GFP-transgenic pig cells. (A) A degenerate embryo on Day-1. (B) An arrested embryos on Day 2 exhibiting un-extruded chromosomes within ooplasm, as indicated by arrows.

In the first experiment, we firstly used GFP/HO-1 transgenic fibroblasts to establish the technical details of the SCNT protocol. Results in Table 4 indicate a high lysis/death rate (40.3±8.0%). Furthermore, the presence of residual chromosomes was confirmed by Hoechst 33342 staining (Figure 5), revealing that these Day 1 reconstructed embryos exhibited incomplete enucleation rather than simple mechanical damage. Similarly, at Day 2, 25.4±3.3% of reconstructed embryos (Table 4) remained arrested at the 1-cell stage (Figure 5) and appeared to retain un-expelled nuclei.

To re-evaluate this phenomenon, we utilized a second fibroblasts of mini-4, which was previously used to clone mini-pigs via handmade cloning [15]. Although the rates of lysis/death and 1-cell stage arrest decreased to 37.6±6.3% and 17.1±3.7%, respectively, these differences were not statistically significant (Table 4). Notably, embryo development to the 2–4 cell stage by Day 2 (82.1±3.4% vs. 69.7±2.5% for L20-01; p<0.05) and the blastocyst stage by Day 6–7 also showed improvement (9.4±3.9% vs. 5.4±3.7% for L20-01; p>0.05). In this experiment, we also attempted to mitigate mechanical damage using 5 μg/mL CB, 1 mM 6-DMAP, and 5 μM Y27632 (a ROCK inhibitor for anti-apoptosis); however, the lysis/death rate remained excessively high (>37.6±6.3%) regardless of the fibroblasts used.

In the second experiment, we switched the donor cells to L277-10 fibroblasts [16] and returned to a 6-DMAP concentration of 2 mM. We compared two treatment windows for 6-DMAP: EBA and EAA. At Day1 and Day 2, the status of the reconstructed embryos was compared between groups. Results (Table 5) showed that the lysis/death rate and the incidence of 1-cell arrest were significantly reduced (p<0.05) from 31.6±4.0% (EBA) to 17.8±4.9% (EAA) and from 19.0±4.6% (EBA) to 7.0±1.0% (EAA), respectively. Furthermore, the rate of 2 to 4-cell embryos was significantly improved (p<0.05) from 73.4±4.7% (EBA) to 91.0±1.4% (EAA).

### Development of L277-10 reconstructed blastocysts

**Figure 6:**
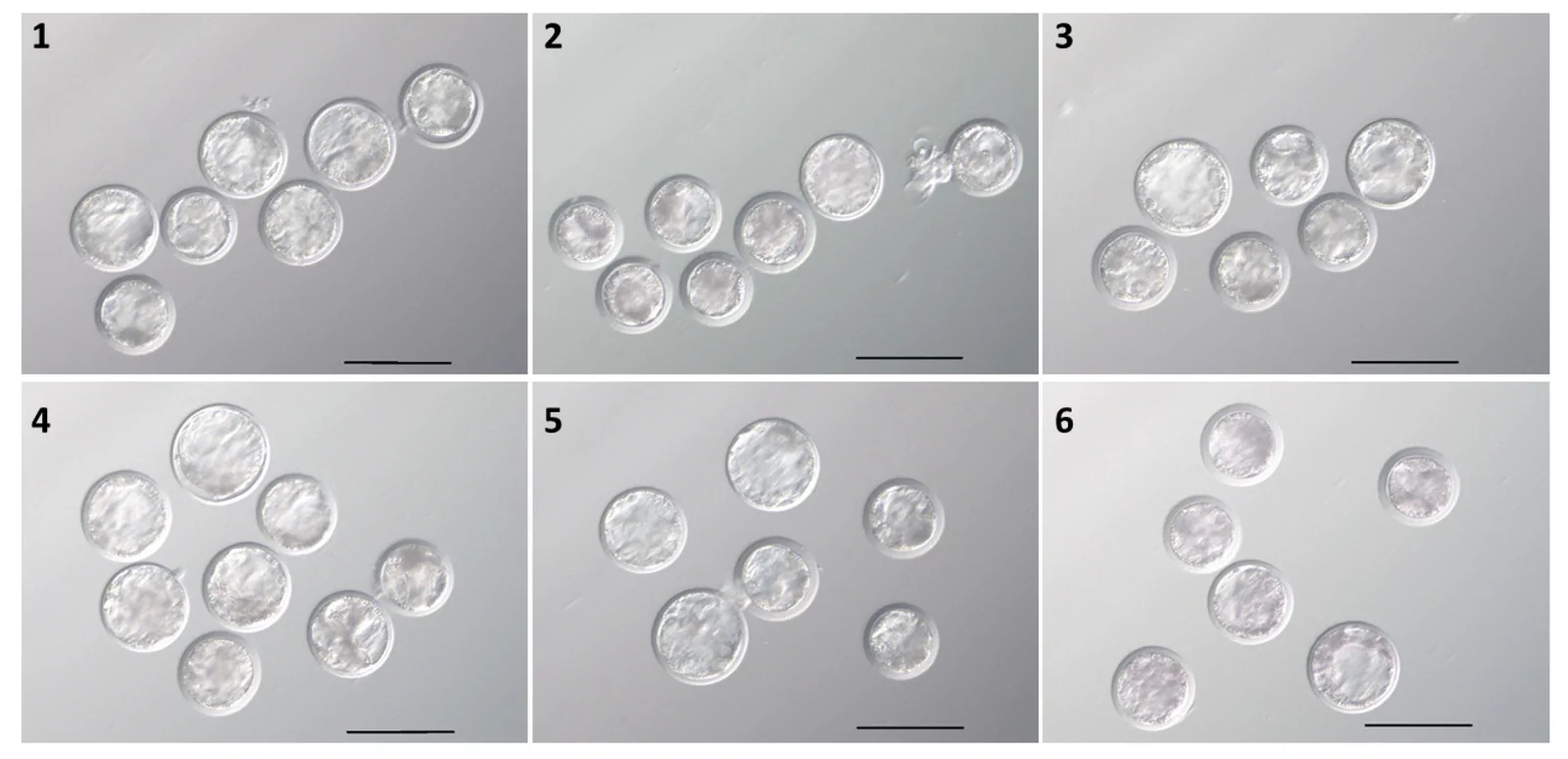
Development of Day 6 reconstructed porcine blastocysts produced using chemically enucleated oocytes and direct microinjection of elite boar somatic cells. These embryos represent the first lot of data shown in Table 6, comprising six independent replicates (1–6). Scale bars represent 200 μm.

By using L277-10 donor cells and the optimized procedures—comprising Noc/LPA-mediated CE, direct microinjection of donor cell into CE ooplasms, 2 h of recovery, Ion/TPEN activation, 6-DMAP treatment for 4 h, and chaetocin treatment for 20–24 h followed by IVC in PZM-5 medium—we evaluated BC formation. Across replicates using approximately 300 ooplasms, we achieved BC formation rates ranging from 9.5±1.3% (n = 25) to 16.6±0.9% (n = 40; Figure 6) after 6 days of IVC (Table 6). By using Day 7 IVC BC, we evaluate the cell number of reconstructed BC and the results revealed 27 to 52 cells (Supplement 4).

## DISCUSSION

SCNT is a cornerstone reproductive technology for generating precisely gene-edited elite pigs, bridging the gap from laboratory research to farm application [1]. However, the production of cloned pigs remains labor-intensive and inefficient, with only approximately 0.3% to 4.3% of transferred embryos successfully developing into live piglets [18]. To facilitate the transition from laboratory research to large-scale farm implementation, this study aimed to simplify the SCNT protocol by utilizing CE—rather than relying solely on CAE—and by implementing the direct microinjection of the donor cells into the CE ooplasm.

By utilizing *in vivo*-matured oocytes and mechanical enucleation, we previously established a direct microinjection method for *in vivo* ooplasm and successfully generated live cloned pigs [16,19]. In this study, we transitioned to IVM oocytes and established a protocol incorporating Noc/LPA-induced CE followed by direct donor cell microinjection.

The use of Ion/Dem to assist enucleation was first reported in rabbit oocytes [5], which later led to generation of live cloning piglets [6]. In those trials, oocytes were treated with Ion for 5 min followed by a 2-h Dem treatment [5,6]. Kawakami et al. [8] further shortened the Dem or Noc treatment time to 1 h without Ion pre-activation, achieving porcine blastocyst developing rates comparable to the control group. While mechanical aspiration enucleation can achieve high success rates (92%–95%) and produce cloned piglets, CAE often requires manipulating the oocytes twice [8]. By preselecting MII oocytes, we established a Noc/LPA-mediated enucleation protocol that enables the direct microinjection of the donor cell in a single and efficient procedure.

During oocytes maturation and the emission of the first polar body, the arrested chromosomes are located near the oocyte membrane beneath an actin cap [20]. We proposed that the efficacy of Noc or Dem treatment for enucleation can be enhanced by LPA, which promotes actin assembly to facilitate the extrusion of the entire chromosomes set. LPA is the simplest glycerophospholipids and a multifunctional phospholipid messenger [21] that regulates mammalian reproduction [22,23]. LPA acts as an activator of RhoA/ROCK signaling pathway—primarily involved in actin dynamics and cell contractility [24]—and organizes the actin cytoskeleton [14] or remodels the formation and dissociation of complexes between actin and actin-biding proteins [25].

In this study, we selected IVM MII oocytes that had successfully emitted the first polar body and treated them with Noc combined with varying concentrations of LPA. The results revealed that 25 μM LPA promoted actin formation around the entire chromosomes set in 88.7±4.2% of oocytes, exhibiting strong or weak actin signals, while 11.3±4.2% of oocytes remained at the MII stage (without visible actin signals). Although oocytes showed spindle reappearance 1 h after removal of Noc/PLA, 83.9±7.3% and 88.1±7.4% of oocytes retained their chromosomes enclosed by actin circle after 1 h and 2 h washout, respectively. Furthermore, we found the spindle reformed as early as 0.5 h washout even enclosed in actin ring (Supplement 3). Consequently, we prepared batches of approximately 50 oocytes per Noc/LPA treatment to ensure that micromanipulation was completed within 30 min of reagent removal.

Furthermore, while activation with Ion might improve enucleation, we omitted Ion activation treatment before or during Noc/LPA enucleation to avoid desynchronization between the donor cell and ooplasm activation. Similar phenomena were reported by Costa-Borges et al. [26], who used ethanol pre-activation and Noc-induced enucleation in mouse oocytes. Their protocol, followed by direct microinjection of the donor cell and a second activation with SrCl_2_ for further 6 h, resulted in poor development to 2-cell or blastocyst stage and failed to produce even cloned fetuses [26].

In porcine model, LPA has been shown to improve oocyte IVM [27,28], accelerate *in vitro* fertilization (IVF) [27,28], and enhance the development of parthenogenetic embryos to the blastocyst stage [27–30]. Most recently, LPA was noted to improve both the quantity and quality of SCNT-derived BC [31]. Here, we report for the first time that LPA enhances Noc-mediated enucleation of MII oocytes via actin formation, thereby facilitating a streamlined SCNT protocol through direct donor cell microinjection into the CE ooplasm.

In SCNT experiment, fibroblasts from a GFP/HO-1 transgenic piglet were used to establish initial parameters of the protocol. After microinjecting the donor cells, reconstructed embryos were recovered for 1.5 to 3 h in culture medium before activation and subsequent 4 h 6-DMAP treatment [15,16]. To shorten the workflow, we tested adding 6-DMAP to the culture medium during the 3 h recovery period. Our results indicate that the rates of lysis or death of reconstructed embryos reached 40% by Day 1. Furthermore, 25% of the reconstructed embryos remained arrested at the 1-cell stage by Day 2. Fluorescence staining revealed that partially or completely un-extruded oocyte chromosomes persisted in the ooplasm. Consequently, we re-evaluated this protocol using donor cells from a female mini pig, which had previously been used to successfully generate live pigs via handmade cloning [15]. Although the development rate to the 2- to 4-cell stage significantly improved, the BC development rate and BC cell number still require further improvement.

These results prompted us to re-evaluate the timing of 6-DMAP treatment. 6-DMAP acts as a protein kinase inhibitor, by inactivating MAPK, that suppresses MPF activity through the inhibition of its catalytic subunit, p34^cdc2^ (also known as CDK1) [32,33]. 6-DMAP has been successfully utilized in ovine [34], bovine [35,36], and porcine [37] SCNT embryos following electrical or chemical activation, resulting in development to term. Standard protocols typically employ a concentration of 1.9 to 2.0 mM for 3 to 4 h. In our experiment with L20-01 and mini-4 fibroblasts, we used 1 mM 6-DMAP combined with 5 μg/mL CB and 5 μM Y27632 (a ROCK inhibitor used to prevent apoptosis) during a 3-h recovery period to minimize mechanical damage and apoptosis. However, the outcomes were unexpectedly poor.

To investigate this further, we compared two 6-DMAP (2.0 mM) treatment schedules using L277-10 fibroblasts [16]: treatment during 3-h recovery period before activation (EBA) versus treatment for 4 h following a 2-h recovery and chemical activation (EAA). Results (Table 5) demonstrated that the EAA group (conventional treatment after activation) showed significant improvements in the lysis/death rate (Day 1), a reduction in the percentage of embryos arrested at the 1-cell stage (Day 2), and an increase in those developing to the 2- to 4-cell stage (Day 2). These findings indicate that 6-DMAP treatment cannot be shortened by combining it with the recovery period, as its function as a protein kinase inhibitor is required post-activation [38]. However, shortening the recovery time itself to 2 h is feasible depending on the cell source [19]. Furthermore, while the 6-DMAP treatment duration showed no difference between 2 and 4 h in canine cloning [39], it might potentially be reduced to 2 or 3 h in porcine cloning. Although L277-10 cells previously yielded eight live pigs via mechanical enucleation of *in vivo* oocytes [16], the blastocyst developing rates using the protocol (9.5%–16.6%) require further improvement. We observed that a substantial number of reconstructed embryos were arrested at 4-cell stage.

## CONCLUSION

While current protocol achieves a BC development rate of 12.6%, several strategies are proposed for further improvement: 1) Donor cell quality: selecting cells with verified normal chromosomal numbers; 2) Oocyte source: utilizing *in vivo*-matured oocytes rather than *in vitro* counterparts to enhance developmental competence; and 3) Culture condition optimization: refining the culture medium—for example, via supplementation with antioxidants [40]—to better support early embryonic development.

In conclusion, supplementation with LPA in combination with Noc enhances chemically induced enucleation in porcine MII oocytes, facilitating the generation of reconstructed BC via the direct microinjection of donor cells into the CE ooplasm. Prospectively, achieving the birth of live pigs is required to definitively demonstrate that CE oocytes are a practical and efficient substrate for SCNT and advanced gene-editing applications in pig breeding.

## Figures and Tables

**Table 1:** Fluorescence intensity of ooplasmic actin signals in matured pig metaphase II (MII) oocytes following treatment with nocodazole (Noc) and various concentrations of lysophosphatidic acid (LPA)

Treatment1)			No. of oocytes	%, oocytes with actin signal2)			% of MII oocytes2)
	
Group	Noc	LPA (μM)		Strong	Weak	Sum	
1.	0	0	50	8.9±3.4^b^	3.5±2.1^b^	12.5±5.0^c^	87.7±5.1^b^
2.	+	0	79	55.6±8.7^a^	22.8±4.9^a^	78.4±5.0^ab^	21.6±5.0^a^
3.	+	10	79	54.3±5.7^a^	22.9±6.0^a^	77.2±6.1^ab^	22.8±6.1^a^
4.	+	25	80	60.2±5.5^a^	27.4±3.1^a^	87.6±3.8^a^	11.3±4.2^a^
5.	+	50	79	39.2±1.8^a^	25.5±5.6^a^	64.7±4.5^b^	35.3±4.5^a^

1) After 44–46 h of IVM, MII oocytes were cultured for 60 min in Ca^2+^ and Mg^2+^ free D-PBS containing various concentrations of LPA and 3 μg/mL Noc.

2) Data are presented as mean±SEM from 6 replicates.

a,b Different letters indicate significant differences (p<0.05).

IVM, *in vitro* maturation; SEM, standard error of the mean.

**Table 2:** Persistence of the actin signal and recovery rates of meiotic spindles following the removal of nocodazole (Noc) and lysophosphatidic acid (LPA) in matured pig metaphase II (MII) oocytes

Treatment1) (n)2)	Oocyte number	Actin signal3)			MII Oocyte3)	Spindle appeared3)	
	
		Strong	Weak	Sum		+ (%)	− (%)
Control (4)	33	3.1±3.1^b^	5.6±3.3^b^	8.8±5.9^b^	91.3±5.9^a^	100.0±0.0^a^	0.0±0.0^c^
1 h (4)	50	61.4±5.1^a^	22.0±1.2^a^	83.9±7.3^a^	16.1±7.3^b^	59.0±21.1^b^	41.0±21.1^b^
2 h (4)	52	48.1±9.5^a^	40.1±16.1^a^	88.1±7.4^a^	11.9±7.4^b^	55.5±17.8^b^	44.5±17.8^b^
4 h (4)	51	53.8±11.8^a^	22.3±6.6^a^	76.1±6.1^a^	23.9±6.1^b^	63.1±22.0^b^	40.7±23.1^b^
Noc/0 h (3)	33	46.5±10.7^a^	10.4±5.8^a^	56.9±10.7^a^	43.1±10.7^b^	0.0±0.0^c^	100.0±0.0^a^

1) Control: oocytes without Noc and LPA treatment. Noc/0 h: immediately after Noc (only and without LPA) treatment; others (1 h, 2 h, and 4 h): oocytes treated with Noc/LPA for 1 h, followed by the removal of agents and a recovery period of 1, 2 or 4 h, respectively.

2) Number of independent experiments.

3) Data are presented as mean±SEM from independent replicates. Data were analyzed by one-way ANOVA followed by Tukey’s multiple comparison test except for spindle formation, which was analyzed using Binomial GLM followed by Fisher’s exact test.

a–c Different letters in the same column indicate significant differences (p<0.05).

SEM, standard error of the mean.

**Table 3:** Effects of ionomycin (Ion) and/or cycloheximide (CHX) activation on enhancing the efficiency of demecolcine (Dem)- or nocodazole (Noc)-mediated enucleation in combination with lysophosphatidic acid (LPA) treatment in matured pig metaphase II (MII) oocytes

Treatment1)					Oocytes number	%, Actin signal2)			%, MII3) oocytes
	
Group	Dem	Noc	Ion	CHX		Strong	Weak	Sum	
1	+	−	−	−	74	60.9±5.9^a^	16.0±2.3^a^	76.8±5.6^a^	23.2±5.6^b^
2	−	+	−	−	74	57.3±6.0^a^	21.1±6.3^a^	78.5±2.1^a^	21.5±2.1^b^
3	+	−	−5	−	77	41.6±5.8^a^	28.0±4.7^a^	69.6±4.1^a^	30.4±4.1^b^
4	−	+	−5	−	77	64.3±3.8^a^	15.9±4.0^a^	80.2±5.0^a^	19.8±5.0^b^
5	+	−	60	−	76	56.9±5.7^a^	14.0±3.6^a^	70.9±4.8^a^	29.1±4.8^b^
6	−	+	60	−	77	53.0±5.9^a^	13.7±6.1^a^	66.8±5.0^a^	33.2±5.0^b^
7	+	−	60	60	74	65.8±7.9^a^	17.9±3.6^a^	83.7±6.0^a^	16.3±6.0^b^
8	−	+	60	60	78	66.9±3.6^a^	18.0±2.9^a^	85.0±2.1^a^	15.0±2.1^b^
9	−	−	−	−	24	4.8±4.83),^b^	0.0±0.0^b^	0.0±0.0^b^	95.2±4.8^a^

Chemical enucleation reagents included 0.4 μg/mL Dem or 3 μg/mL Noc, 25 μM LPA, 15 μM Ion, and 10 μg/mL CHX. Ion/-5 indicates MII oocytes were activated with Ion for 5 min, followed by treatment with Dem or Noc combined with LPA for 60 min. Ion/60 indicates oocytes were treated with Dem or Noc combined with Ion and LPA for 60 min. Ion/60+CHX/60 indicates treatment with Dem or Noc combined with LPA, Ion and CHX for 60 min. All MII oocytes were maturated *in vitro* for 43–46 h; the enucleation medium was Ca^2+^- and Mg^2+^-free D-PBS.

1) n = 7.

2) Data are presented as means±SEM.

3) One oocyte exhibited a strong actin signal.

a,b Different letters in the same column indicate significant differences (p<0.05). One-way ANOVA followed by Tukey’s multiple comparison test was used.

SEM, standard error of the mean.

**Table 4:** Developmental rates of reconstructed embryos generated by direct somatic cell nuclear transfer (SCNT) into oocytes chemically enucleated with nocodazole and lysophosphatidic acid

Somatic cells (5)1)	No. (%) of oocytes2)		No. (%) of developed2)					
	
	Total	Lysis/death	Total	Day 2		Day 6–7		
	
				1C	2–4C	Blastocysts (BC)3)		Cell number of BC
L20-01	258	103 (40.3±8.0)	155	39 (25.4±3.3)	107 (69.7±2.5)^a^	5	(5.4±3.7)	20.2±4.5
Mini-4	250	94 (37.6±6.3)	146	24 (17.1±3.7)	121 (82.1±3.4)^b^	15	(9.4±3.9)	23.3±2.0

Oocytes were treated with 3 μg/mL nocodazole and 25 μM lysophosphatidic acid for 1 h. Following SCNT, the reconstructed embryos were recovered for 3 h in medium supplemented with 1 mM 6-DMAP, 5 μg/mL cytochalasin B (CB), and 5 μM Y27632, followed activation and *in vitro* culture.

1) Numbers of SCNT replicates.

2) Mean±SEM.

3) Three replicates of L20-01 (GFP/hHO-1 transgenic) and two replicates of Mini-4 SCNT failed to develop to the BC stage.

a,b Different letter in the same column indicate significant differences (p<0.05).

SEM, standard error of the mean.

**Table 5:** Effects of 6-DMAP treatment timing on Day 2 development of SCNT embryos produced via direct microinjection of the donor cells into oocytes chemically enucleated with nocodazole and lysophosphatidic acid

6-DMAP (n)	Reconstructed embryos at Day 1			IVC, developing at Day 2				

	Total	No. (%) of lysis/death1)		Sub-total	1-Cell		2 to 4-Cell	
	
					No.	(%)1)	No.	(%)1)
EBA2) (8)	397	125	(31.6±4.0)	272	52	(19.0±4.6)	200	(73.4±4.7)
EAA2) (5)	248	44	(17.8±4.9)	204	14	(7.0±1.0)	186	(91.0±1.4)
p-value3)			0.046			0.044		0.011

Treated with 3 μg/mL nocodazole and 25 μM lysophosphatidic acid for 1 h.

1) Mean±SEM.

2) EBA and EAA indicate embryos treated with 6-DMAP before and after activation, respectively.

3) t-test.

SCNT, somatic cell nuclear transfer; IVC, in vitro culture; EBA, embryo-treated before activation; EAA, embryo-treated after activation.

**Table 6:** Developmental rates of SCNT embryos produced via direct microinjection of donor cells (L277-10) into chemically enucleated oocytes by nocodazole and lysophosphatidic acid treatment

Lot1)	Reconstructed embryos at Day 1			IVC, developing at Day 2					BC, developing at Day 6	

	Total	No. (%) lysis/death2)		Sub-total	1-Cell		2 to 4-Cell		No.	(%)2)
	
					No.	(%)2)	No.	(%)2)		
1	298	55	(18.4±3.7)	243	27	(9.1±1.9)	210	(86.2±1.8)	40	(16.6±0.9)
2	300	36	(12.0±1.8)	264	26	(8.7±1.3)	238	(90.2±1.5)	25	(9.5±1.3)
3	297	60	(16.9±2.5)	237	52	(17.5±3.3)	182	(77.2±2.9)	28	(11.7±1.9)
Total	895	151	(16.9±1.7)	744	105	(11.7±2.3)	6303)	(84.5±2.5)	93	(12.6±1.5)

Treated with 3 μg/mL nocodazole and 25 μM lysophosphatidic acid for 1 h.

1) Each lot was repeated 6 times.

2) Mean±SEM.

3) Excluding fragmented embryos that superficially resembled morula or displayed advanced degeneration.

SCNT, somatic cell nuclear transfer; IVC, *in vitro* culture; BC, blastocysts.

## SUPPLEMENTARY MATERIAL

**Supplement 1.** Enucleation rates (%) following 60 min treatment with demecolcine and nocodazole.

**Supplement 2.** Using lysophosphatidic acid (LPA) potentiates demecolcine (Dem) to enucleate IVM metaphase II (MII) oocytes.

**Supplement 3.** Spindle reformation 0.5 h after Noc/LPA washout.

**Supplement 4.** Reconstructed blastocysts produced via direct microinjection of L277-10 donor cells into chemically enucleation cytoplasm.
