# Supplementary material for: Lysophosphatidic acid-mediated enhancement of porcine oocyte chemical enucleation for somatic cell nuclear transfer
Source: Anim Biosci. 2026 Jun 1;39(9):260269. doi: 10.5713/ab.260269 (PMC13547751; doi:10.5713/ab.260269)
Supplement: Supplementary file 1 [file ab-260269-Supplementary-1.pdf]

Supplement 1. Enucleation rates (%) following 60 min treatment with demecolcine and nocodazole

	Lot			
Treatment	I	II	III	Sum (%)
Demecolcine, 0.4 μg/mL	22/30[1]<br>(73.3)	45/50<br>(90.0)	21/28<br>(75.0)	88/108<br>(79.4 ± 5.3)
Nocodazole, 3 μg/mL	21/28<br>(75.0)	27/28<br>(96.4)	30/40<br>(75.0)	78/ 96<br>(82.1 ± 7.1)

[1] Number of enucleated oocytes/total observed oocytes; all oocytes were stained with Hoechst 33342.
