# Supplementary material for: Lysophosphatidic acid-mediated enhancement of porcine oocyte chemical enucleation for somatic cell nuclear transfer
Source: Anim Biosci. 2026 Jun 1;39(9):260269. doi: 10.5713/ab.260269 (PMC13547751; doi:10.5713/ab.260269)
Supplement: Supplementary file 2 [file ab-260269-Supplementary-2.pdf]

Supplement 2. Using lysophosphatidic acid (LPA) potentiates demecolcine (Dem) to enucleate IVM metaphase II (MII) oocytes[1)]

Treatment (n=4)			No. of oocytes	% of oocytes with actin signal[2)]			% of MII oocytes[2)]
Group	Dem	LPA, µM		Strong	Weak	Sum	
1.	0	0	28	10.7 ± 6.0$^{a, 2}$	14.6 ± 1.2	24.0 ± 4.9^{a}	76.0 ± 4.9^{b}
2.	+	0	42	44.2 ± 9.3^{b}	27.0 ± 8.1	71.4 ± 4.0^{b}	28.8 ± 4.0^{a}
3.	+	10	51	48.6 ± 7.7^{b}	29.9 ± 4.7	78.4 ± 4.7^{b}	21.6 ± 4.7^{a}
4.	+	25	48	53.9 ± 5.8^{b}	25.1 ± 4.8	78.9 ± 3.0^{b}	21.1 ± 3.0^{a}
5.	+	50	49	59.8 ±8.3^{b}	14.4 ± 5.3	74.2 ± 6.0^{b}	25.8 ± 6.0^{a}

[1)] After 43 h IVM, the MII oocytes being cultured with LPA and 0.4 µg/mL Dem in D-PBS (Ca^{2+} and Mg^{2+} free) for 60 min.

[2)] Data are presented as mean ± SEM from four independent replicates. Different letters indicate significant differences ($p < 0.05$).
