# Supplementary material for: Lysophosphatidic acid-mediated enhancement of porcine oocyte chemical enucleation for somatic cell nuclear transfer
Source: Anim Biosci. 2026 Jun 1;39(9):260269. doi: 10.5713/ab.260269 (PMC13547751; doi:10.5713/ab.260269)
Supplement: Supplementary file 3 [file ab-260269-Supplementary-3.pdf]

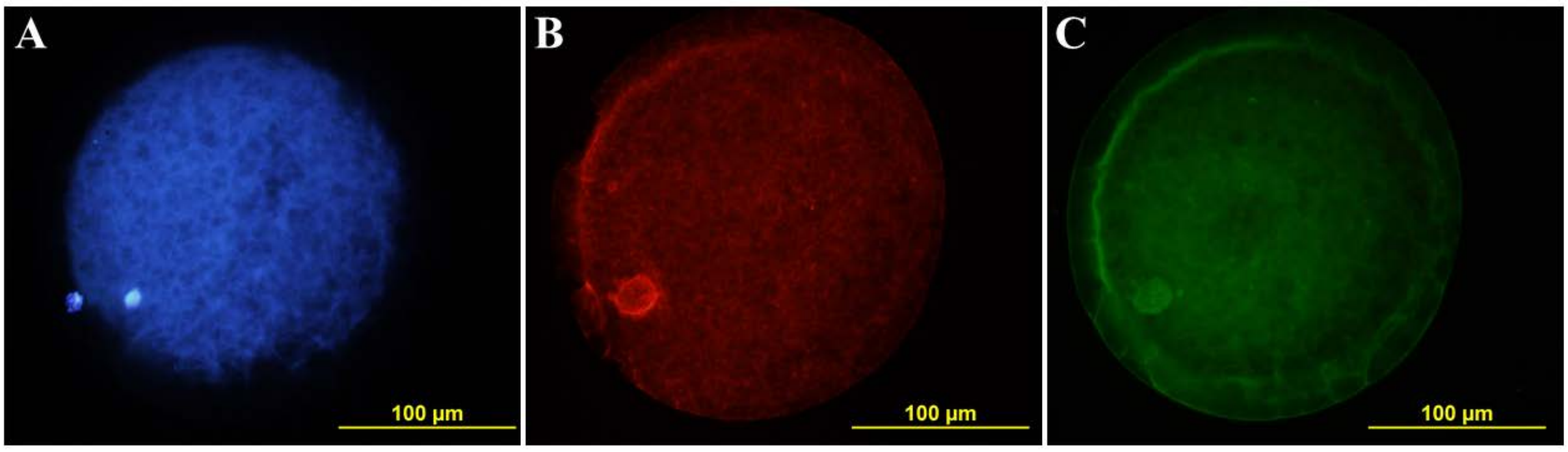

Supplement 3. Spindle reformation 0.5 h after Noc/LPA washout. **(**A) DNA stained with Hoechst 33342; (B) Actin staining; and (C) Meiotic spindle staining.
