# Supplementary material for: Lysophosphatidic acid-mediated enhancement of porcine oocyte chemical enucleation for somatic cell nuclear transfer
Source: Anim Biosci. 2026 Jun 1;39(9):260269. doi: 10.5713/ab.260269 (PMC13547751; doi:10.5713/ab.260269)
Supplement: Supplementary file 4 [file ab-260269-Supplementary-4.pdf]

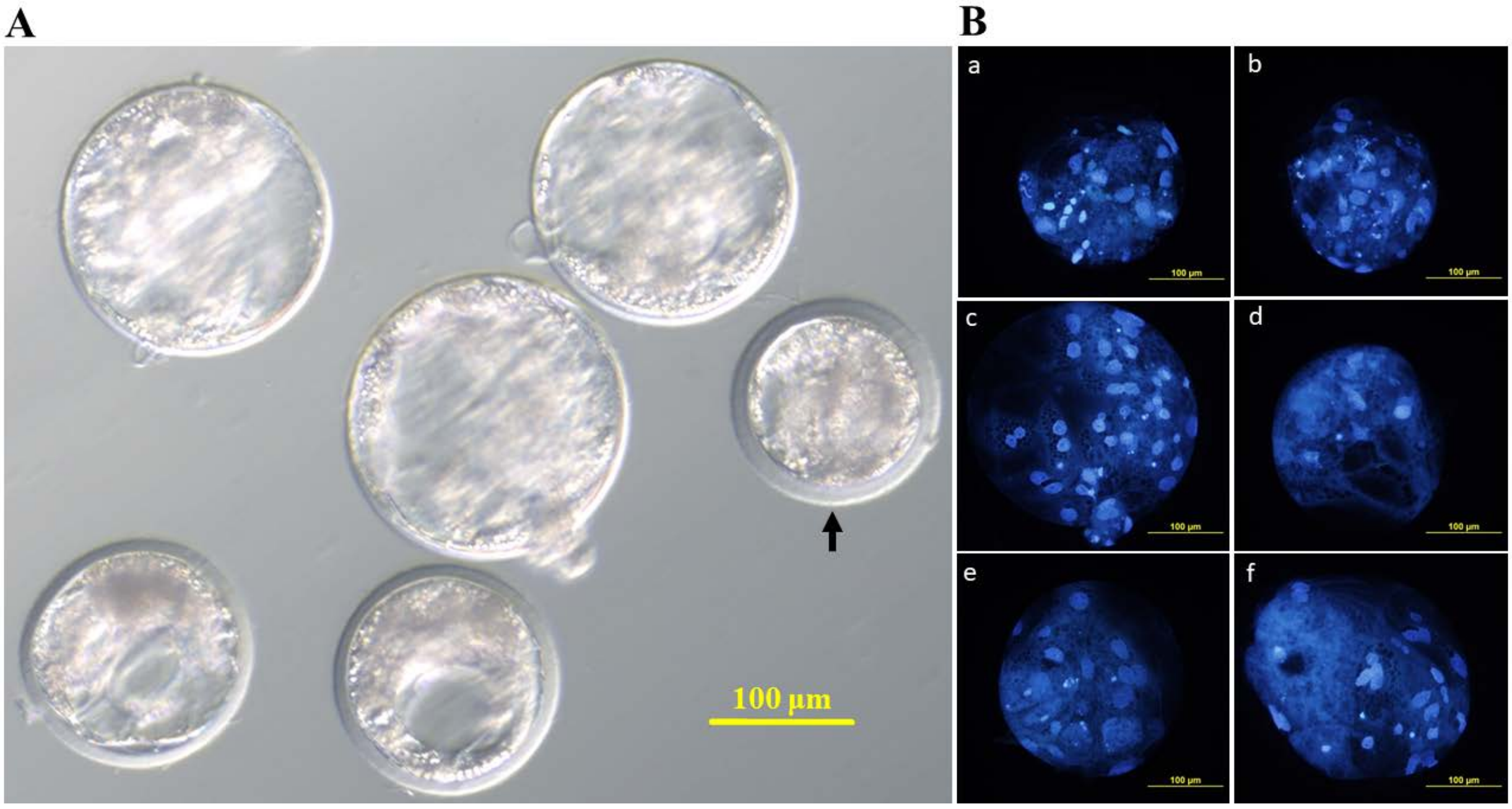

Supplement 4. Reconstructed blastocysts produced via direct microinjection of L277-10 donor cells into chemically enucleation cytoplasm. (A) Day 7 reconstructed embryos; the embryo indicated by black arrow (B-d) has not reached the blastocyst stage. (B) Blastocysts were validated by Hoechst 33342 staining and reveal the cell counts.
